# Tracking tumor kinetics in patients with germline *CYLD* mutations

**DOI:** 10.1016/j.jaad.2018.04.014

**Published:** 2018-11

**Authors:** Sarah Brown, Sylvia A. Worthy, James A.A. Langtry, Neil Rajan

**Affiliations:** a Department of Dermatology, Royal Victoria Infirmary, Newcastle Upon Tyne, United Kingdom; b Department of Radiology, Royal Victoria Infirmary, Newcastle Upon Tyne, United Kingdom; c Institute of Genetic Medicine, Newcastle University, Newcastle upon Tyne, United Kingdom

*To the Editor:* Objective data on the rate of growth of skin tumors in the rare orphan disease CYLD cutaneous syndrome (CCS; synonym Brooke-Spiegler syndrome) are lacking. Its clinical burden on patients is significant, warranting the clinical trial of personalized treatments. To inform the design of trials of novel therapies, natural history studies that capture the rate of tumor growth in CCS are needed. The absence of such data on this rare genetic disease prompted our work to gather opportunistic tumor measurements from radiologic investigations carried out in patients with CCS as part of their routine clinical care.

Here we report the rate of tumor growth in 3 patients with CCS who underwent serial computed tomography (CT) imaging for monitoring pulmonary cylindromas^1^, ^2^ or malignant tumors. Four individuals were identified as having undergone CT imaging following a retrospective case note and radiologic data review of 16 CCS patients attending a tertiary dermatogenetics center, 3 of whom had CT-detectable tumors.^3^, ^4^ These 3 females had been documented to carry a familial heterozygous mutation in the *CYLD* gene (c.2460delC).^5^ They had features of a severe phenotype, including a history of complete scalp excision in 2 of them. Ethical approval to study these patients (who had given consent) was obtained from a research ethics committee.

In each patient, tumors were located and numbered on a baseline high-resolution CT scan. Each tumor was measured on every slice on which it was visible, after which the longest diameter measured was chosen for each time point. This axis of measurement was maintained on subsequent imaging. We elected to exclude tumors smaller than 3 mm, lesions seen on only 1 slice, confluent tumors, and tumors arising in scar tissue (as this tissue was often isodense to the tumor) to allow for maximal accuracy and reproducibility across all scans. Data from new lesions detected during interval scans were included from the point of first appearance and tracked in subsequent scans. Excised lesions for which more than 1 time point was measured were tracked to the point immediately before excision; tumors that were seen at only 1 measurement time point were excluded, as they did not contribute to the rate of change data. Relative changes in size were calculated by comparing measurements at the indicated time points against baseline. Patients were followed over a mean period of 495.6 days (range, 226-891 days).

**Fig 1:**
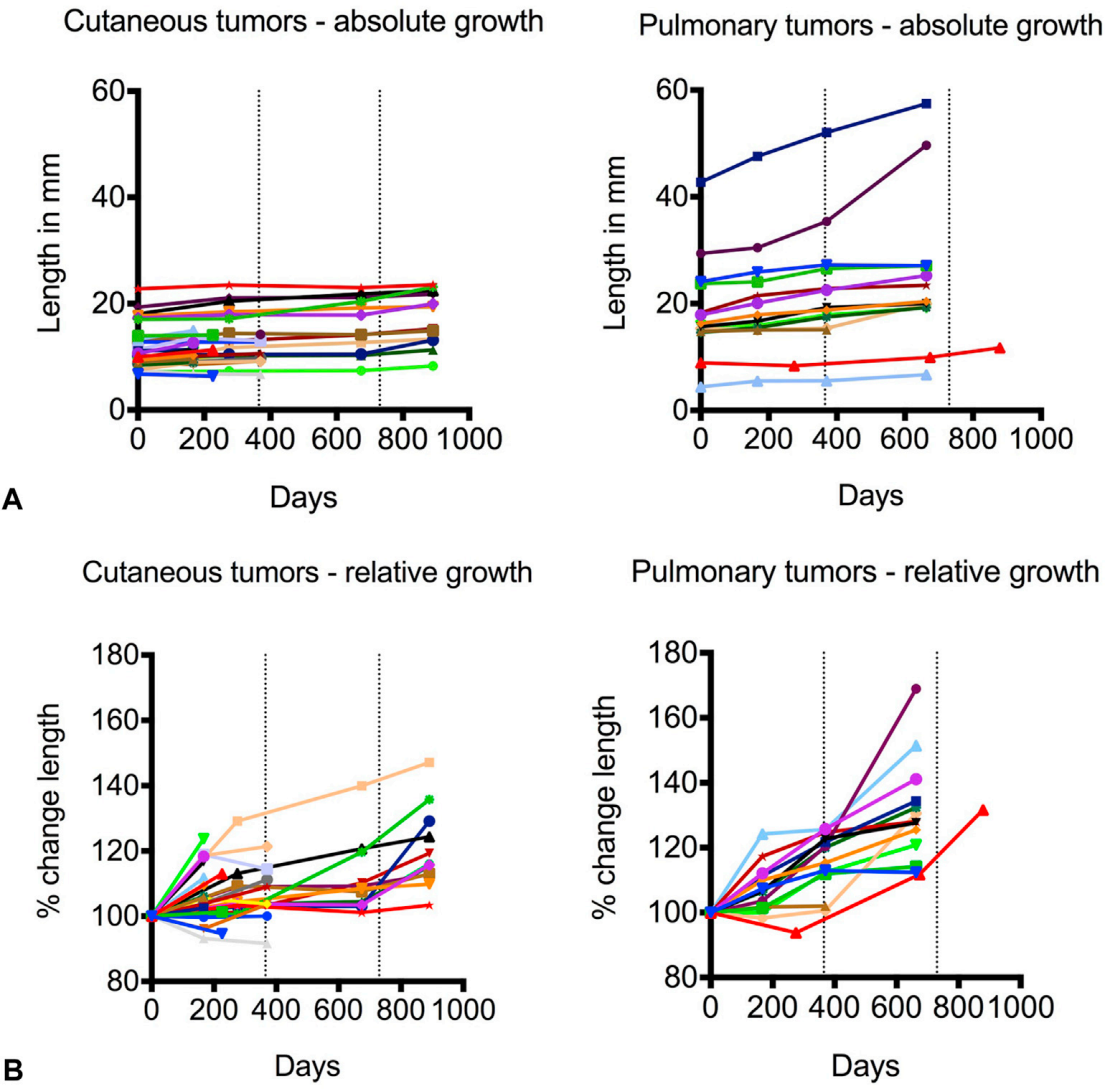
Cylindroma. Growth kinetics of cutaneous and pulmonary tumors. **A,** Line graph indicating changes in the longest dimension (in mm) displayed over time for cutaneous and pulmonary tumors. **B,** Line graph showing tumor growth kinetics as percentage change in the longest dimension compared with baseline over time in cutaneous and pulmonary cylindromas. (Dotted lines on *x* axis indicate 1- and 2-year intervals.)

Of the cutaneous cylindromas, 30 grew and 2 decreased in size during the period of observation. The mean size at baseline was 12.6 mm (range, 6.7-23.5 mm) (Fig 1, *A*). The average increase in size of cutaneous tumors was 12.6% (range, –8.7% to 41.1%) per year (Fig 1, *B*). Fourteen pulmonary cylindromas were studied; all of them increased in size. The mean size of the pulmonary tumors at baseline was 18.7 mm (range, 4.4-42.7 mm) (Fig 1, *A*). The average increase in size of the pulmonary tumors was 16.3% (range, 1.1%-38.0%) per year (Fig 1, *B*).

Our findings are important for the following reasons. First, these data may inform routine clinical surveillance intervals, with insights into both cutaneous and pulmonary tumor kinetics. Second, our approach is informative as a proof of principle that serial radiologic imaging can be used to monitor change in the size of cutaneous tumors in this condition. Taken together with anticipated growth rates, this will inform the design and development of meaningful measures of clinical trial outcomes.

Our preliminary data on growth rate is informative; however, caveats that should be considered include patient genotype, body site–specific factors, and excision bias. Nonetheless, our work leverages the multiplicity of lesions seen in these patients and represents opportunistic insight into the tumor kinetics of these rare cutaneous tumors.
